# Wnt/β-catenin-mediated signaling re-activates proliferation of matured cardiomyocytes

**DOI:** 10.1186/s13287-018-1086-8

**Published:** 2018-12-07

**Authors:** Yong Fan, Beatrice Xuan Ho, Jeremy Kah Sheng Pang, Nicole Min Qian Pek, Jin Hui Hor, Shi-Yan Ng, Boon-Seng Soh

**Affiliations:** 1 Key Laboratory for Major Obstetric Diseases of Guangdong Province, The Third Affiliated Hospital of Guangzhou Medical University, Guangzhou, 510150 China; 2 Disease Modeling and Therapeutics Laboratory, A*STAR Institute of Molecular and Cell Biology, 61 Biopolis Drive Proteos, Singapore, 138673 Singapore; 3 Department of Biological Sciences, National University of Singapore, Singapore, 117543 Singapore; 4 Neurotherapeutics Laboratory, A*STAR Institute of Molecular and Cell Biology, 61 Biopolis Drive Proteos, Singapore, 138673 Singapore; 5 National Neuroscience Institute, 11 Jalan Tan Tock Seng, Singapore, 308433 Singapore; 6 Department of Physiology, National University of Singapore, 2 Medical Dr, Singapore, 117593 Singapore

**Keywords:** Wnt signaling, Matured cardiomyocytes, Cardioproliferation, N-cadherin, GSK inhibitor, Embryonic stem cells

## Abstract

**Background:**

The Wnt/β-catenin signaling pathway plays an important role in the development of second heart field (SHF Isl1+) that gives rise to the anterior heart field (AHF) cardiac progenitor cells (CPCs) for the formation of the right ventricle, outflow tract (OFT), and a portion of the inflow tract (IFT). During early cardiogenesis, these AHF CPCs reside within the pharyngeal mesoderm (PM) that provides a microenvironment for them to receive signals that direct their cell fates. Here, N-cadherin, which is weakly expressed by CPCs, plays a significant role by promoting the adhesion of CPCs within the AHF, regulating β-catenin levels in the cytoplasm to maintain high Wnt signaling and cardioproliferation while also preventing the premature differentiation of CPCs. On the contrary, strong expression of N-cadherin observed throughout matured myocardium is associated with downregulation of Wnt signaling due to β-catenin sequestration at the cell membrane, inhibiting cardioproliferation. As such, upregulation of Wnt signaling pathway to enhance cardiac tissue proliferation in mature cardiomyocytes can be explored as an interesting avenue for regenerative treatment to patients who have suffered from myocardial infarction.

**Methods:**

To investigate if Wnt signaling is able to enhance cellular proliferation of matured cardiomyocytes, we treated cardiomyocytes isolated from adult mouse heart and both murine and human ES cell-derived matured cardiomyocytes with N-cadherin antibody or CHIR99021 GSK inhibitor in an attempt to increase levels of cytoplasmic β-catenin. Immunostaining, western blot, and quantitative PCR for cell proliferation markers, cell cycling markers, and Wnt signaling pathway markers were used to quantitate re-activation of cardioproliferation and Wnt signaling.

**Results:**

N-cadherin antibody treatment releases sequestered β-catenin at N-cadherin-based adherens junction, resulting in an increased pool of cytoplasmic β-catenin, similar in effect to CHIR99021 GSK inhibitor treatment. Both treatments therefore upregulate Wnt signaling successfully and result in significant increases in matured cardiomyocyte proliferation.

**Conclusion:**

Although both N-cadherin antibody and CHIR99021 treatment resulted in increased Wnt signaling and cardioproliferation, CHIR99021 was found to be the more effective treatment method for human ES cell-derived cardiomyocytes. Therefore, we propose that CHIR99021 could be a potential therapeutic option for myocardial infarction patients in need of regeneration of cardiac tissue.

**Electronic supplementary material:**

The online version of this article (10.1186/s13287-018-1086-8) contains supplementary material, which is available to authorized users.

## Background

Cardiovascular disease is currently the leading cause of death globally and is expected to rise in future as the incidence and prevalence of chronic diseases associated to cardiovascular risk factors increases [1]. In conjunction with preventive medicine, cardiac regeneration therapy may therefore be the next best solution to overcome this problem. Efforts are now being focused on improving the efficiency of cardiomyocyte proliferation to preserve cardiac function after an injury [2–4]. Hence, understanding the mechanism of cardiovascular development that leads to cardiovascular proliferation is pivotal in the advancement of therapeutic approach aimed at cardiac pathology.

The Wnt/β-catenin signaling pathway in particular plays an important role in the development of second heart field (SHF Isl1+) that gives rise to the anterior heart field (AHF) cardiac progenitor cells (CPCs) for the formation of right ventricle (RV), outflow tract (OFT) and portion of inflow tract (IFT) [5–9]. During early cardiogenesis, these AHF CPCs reside within the pharyngeal mesoderm (PM) that provides a microenvironment for them to receive inputs and direct their fate. N-cadherins are Ca2+ dependent transmembrane proteins that associate with cytoplasmic catenins, such as the Wnt signal transducer β-catenin, to form adherens junction protein complexes which enable intercellular adhesion while simultaneously sequestering β-catenin to the plasma membrane. Unbound β-catenin are able to undergo nuclear translocation, forming complexes with the TCF/LEF family of DNA binding proteins to activate downstream Wnt effector genes, consequently promoting cardioproliferation and preventing premature differentiation of CPCs [5]. As such, cadherins are generally treated as negative regulators of the Wnt signaling pathway. However, N-cadherin, although weakly expressed in AHF CPCs, play a significant role in their cell fate and development by promoting intercellular adhesion between AHF CPCs while also being crucial to maintain high Wnt signaling activity [5]. On the contrary, adult myocardium express N-cadherin strongly, and yet have low levels of Wnt signaling activity and regenerate at a very low frequency, with an estimated rate of 0.5–1% per year [10–12]. We therefore attempted to revert matured cardiomyocytes into a more proliferative state by activating high levels of Wnt signaling. We posit that N-cadherin expression is necessary to protect a cytoplasmic store of β-catenin from proteolytic degradation via the GSK ubiquitination complex. These stores result in high Wnt signaling in CPCs due to the presence of Wnt signaling ligands binding and releasing β-catenin, while in matured cardiomycytes, β-catenin is highly sequestered and do not contribute to Wnt signal transduction. These stores of β-catenin, if released and made available in matured cardiomyocytes, should be able to undergo nuclear translocation and activate Wnt signaling to promote cardioproliferation.

In this study, we demonstrated that re-activation of Wnt/β-catenin signaling pathway can be achieved in matured mouse and human ES cell-derived cardiomyocytes by using N-cadherin antibody or GSK inhibitor, CHIR99021, releasing sequestered β-catenin in the N-cadherin-based adherens junctions or by preventing the ubiquitination-degradation of β-catenin, respectively [13–15]. We further validated the mechanism, demonstrating the translocation of cytoplasmic β-catenin into the nuclei to re-activate Wnt signaling leading to cardioproliferation. This study has therefore provided an avenue for the expansion of matured cardiomyocytes in vitro and perhaps regenerative medicine through enhanced cardiomyocyte proliferation to preserve cardiac function after myocardial injury.

## Methods

### Cell culture of human and murine ES cells for differentiation towards cardiomyocytes

The human ES cell line (H7) was cultured in feeder-free condition on Matrigel (BD, Franklin Lakes, NJ). The cells were maintained in mTeSR™1 medium (Stemcell Technologies) and passaged with collagenase IV (1 mg/ml) enzymatic treatment. To differentiate human ES cells towards cardiomyocytes, we adopted the protocol established by Lian et al. [16, 17]. The *Cdh2* mouse knockout ES cells were cultured and differentiated towards cardiomyocytes as described by Soh et al. [5]. In this study, matured ES cell-derived cardiomyocytes were cultured for more than 2 months from the initial contraction to ensure sufficient cardiomyocyte maturation [18].

### Isolation of human and murine ES cell-derived cardiomyocyte

Single-cell suspension was obtained from cardiomyocytes derived from both murine and human ES cells. The cells were stained using vascular cell adhesion molecule (VCAM-1) and SIRPα/β antibodies, respectively. Briefly, staining of mouse cardiomyocytes was achieved with rabbit anti-VCAM1 monoclonal antibody (1:50) (Cell Signaling Technologies) in the presence of blocking buffer consisting of 5% FBS and 2% BSA in PBS for 90 min at 37 °C, followed by donkey anti-rabbit IgG Alexa Fluor 594 at 1:1000 dilution (Invitrogen) for 1 h. Human ES cell-derived cardiomyocytes, on the other hand, were stained with PE/Cy7-conjugated anti-human CD172a/b (SIRPα/β) antibody at 1:300 dilution (Biolegend). Cardiomyocytes were subsequently purified via fluorescence-activated cell sorting (FACS). Matured human ES cell-derived cardiomyocytes were treated with either 100 μM of TBP or 100 nmol/L of EDN1 to induce cardiac hypertrophy.

### Isolation and culture of matured mouse cardiomyocyte

Matured cardiomyocytes were isolated from mice that are at least 2 months old according to published protocol [19]. The isolated cardiomyocytes were maintained in medium comprising of RPMI and B27 supplement [16].

### RNA isolation and quantitative PCR

For cultured cell samples, 2 × 10^6^ cells were harvested and lysed in 800 μl of TRIzol reagent (Invitrogen). The samples were allowed to stand for 5 min at room temperature, after which 160 μl of chlorofoam was added to allow for phase separation by centrifugation at 12,000×*g* for 15 min at 4 °C. Following that, the aqueous phase was transferred to a fresh tube, and equal volume of isopropanol was added and mixed. RNA samples were allowed to precipitate at room temperature for another 10 min. The precipitated RNA samples were pelleted by centrifugation at 12,000×*g* for 15 min at 4 °C. For cDNA synthesis, RNA samples (500 ng) were reverse transcribed to obtain cDNA using the iScript cDNA Synthesis kit (BioRad). Primer sequences are provided in Additional file 1: Table S1. Quantitative PCR analyses were performed using SYBR Green Master Mix Reagent (Applied Biosystems) on an ABI Viia7 Real-Time PCR System. The threshold cycle (Ct) was determined to be ≥ 35. Each experiment was repeated at least twice. Standard deviations (s.d.) of the means in qPCR experiments were obtained from three independent experiments.

### Immunocytochemistry

Immunocytochemical analysis was performed using the respective antibodies: mouse anti-PY654-β-catenin (membrane bound) and anti-PY489-β-catenin (nuclear) (both from Hybridoma bank) and mouse anti-cardiac troponin T (1:400; Abcam ab8295). Briefly, cells were first harvested and washed once with PBS. Fixation of cells was achieved with 4% paraformaldehyde for 30 min, followed by treatment with 0.3% Triton X to permeabilize cells, if necessary. The cells were then blocked with PBS containing 5% fetal bovine serum and 1% BSA for 30 min at room temperature. Primary antibodies were added at respective dilutions and incubated for 1 h at room temperature. After washing, the cells were incubated for another 45 min at room temperature without light exposure with either 1:1000 diluted Alexa Fluor 488 or Alexa Fluor 594 secondary antibodies. Nuclei were counterstained with 4,6-diamindino-2-phenylindole (DAPI). The cells were observed under a fluorescent microscope (Nikon TS-100).

### Image acquisition and analysis

Fluorescent images were acquired by an automated microscope (PerkinElmer Operetta) at ×20 magnification. Image analysis was performed using the Columbus software (PerkinElmer), by first recognizing and outlining cellular nuclei based on DAPI staining. For each experiment, equal number of SIRPA-positive cardiomyocytes (1 × 10^5^ cells) was seeded to compare proliferation of treated cells (N-cadherin antibody or CHIR99021) vs control (untreated). Quantification of cardiomyocytes was performed by summing all cardiac troponin T (cTnT)-positive cells (relative cutoff intensity > 150) in the treated wells vs the control. For the analysis on cardiomyocyte proliferation, the double-positive cells (Ki67+/cTnT+ or BrdU+/cTnT+) in each of the treatment conditions have been calculated as a percentage of all the single-positive cells (cTnT+).

### Western blot

Whole-cell lysates were generated by scraping cultures of matured cardiomyocytes treated with CHIR99021, N-cadherin antibody, or combination for 1 day and resuspended in RIPA lysis buffer (1% Nonidet P-40, 0.5% deoxycholate, 5 M NaCl, and 1 M Tris pH 7.4) for 10 min at 4 °C and then centrifuged at 11,400*g* for 20 min at 4 °C for the isolation of the cytosolic fractions. Quantification of protein concentration was determined using Pierce BCA Protein Assay kit (Thermo-scientific). Protein lysate of 60 μg per sample was loaded onto 4–20% Mini-PROTEAN TGX Stain-Free Precast Gels (Bio-Rad), resolved by electrophoresis, and transferred to nitrocellulose membrane (Bio-Rad). Membranes were blocked with 5% skimmed milk in TBS + 1% Tween-20 and probed overnight with primary antibodies. The antibodies were rabbit anti-β-catenin antibody (1:2500; Abcam ab32572) and mouse anti-β-actin antibody (1:1000; Cell Signaling). Membranes were washed and incubated for 1 h with their respective IgG-HRP-conjugated secondary antibody (Santa Cruz) in 5% skimmed milk in TBS + 1% Tween-20 and developed using ECL substrate (Bio-Rad).

### Statistics

Results involving two experimental groups expressed as mean ± s.d. were tested for statistical significance using Student’s *t* test, two-sided based on assumed normal distributions. *P* values < 0.05 were considered statistically significant. The error bars in graphs with three or more experimental groups refer to standard error of means of stated replicates. A non-parametric test (Kruskal-Wallis one-way analysis of variance) was used to assess whether the mean values of the experimental groups could be considered to be significantly different compared to the control group at 95% confidence level.

## Results

### Re-activation of Wnt/β-catenin signaling increases matured mouse cardiomyocyte proliferation

**Fig. 1:**
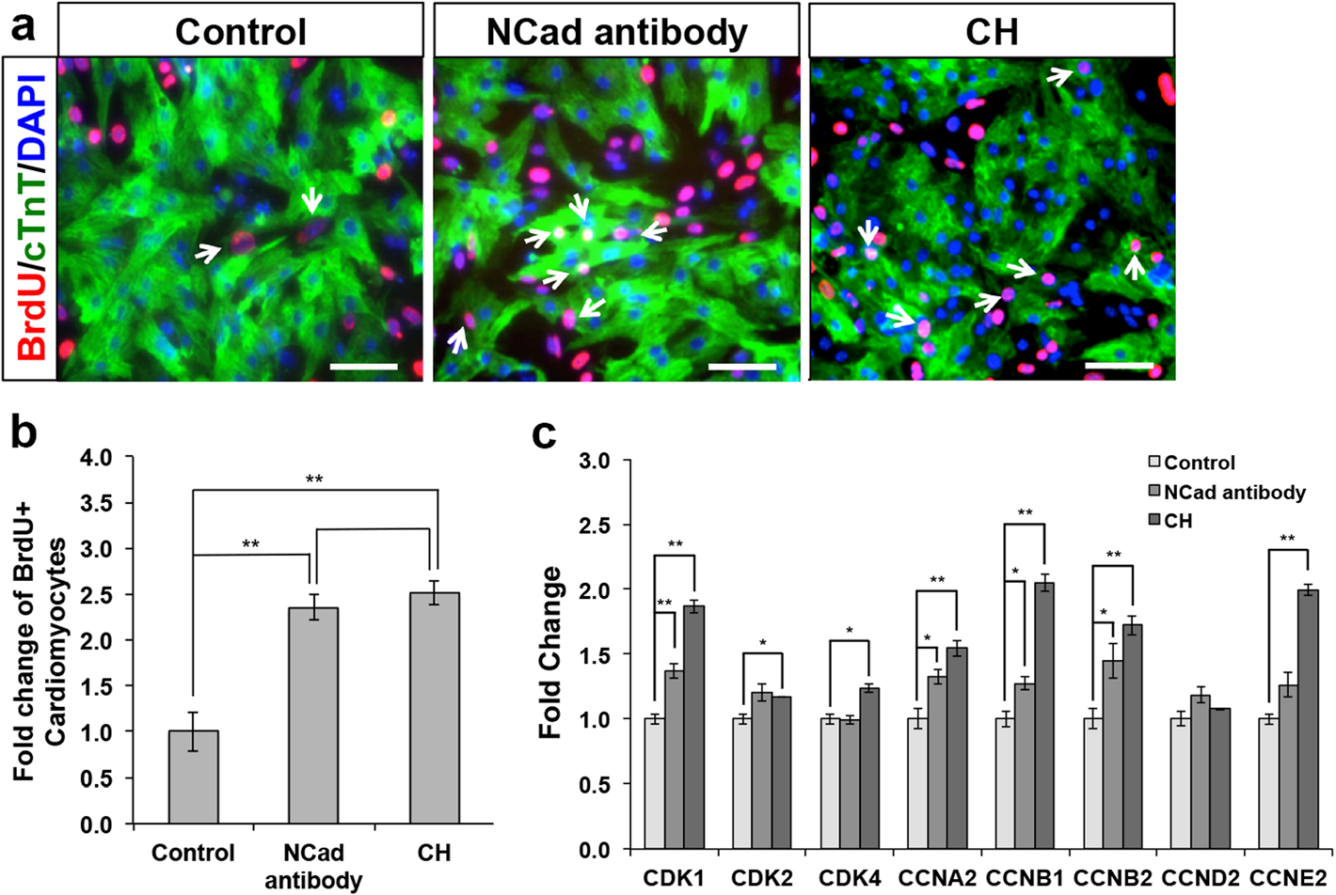
Re-activation of Wnt/β-catenin signaling increases matured mouse cardiomyocyte proliferation. **a** Representative images of untreated control and treated matured mouse cardiomyocytes with either N-cadherin antibody (1:1000) or CHIR99021 (1 μM) immunostained for BrdU (red) and cTnT (green); BrdU and cTnT double-positive cells are marked by white arrows. Cell nuclei (blue) were stained with DAPI. Scale bar, 100 μm. **b** Graphical representation showing fold change in BrdU+ cardiomyocytes in N-cadherin antibody (1:1000) and CHIR99021 (1 μM)-treated cardiomyocytes compared to untreated cardiomyocytes (control). Note the ~ 1.5-fold increase in BrdU-positive cardiomyocytes in treated cardiomyocytes as compared to control. Error bars indicate s.d., *n* = 3 experiments, *P < 0.05 for Kruskal-Wallis one-way analysis of variance compared to control. **c** Quantitative PCR analysis of mRNA isolated from cardiomyocytes from untreated control and cardiomyocytes treated with N-cadherin antibody (1:1000) or CHIR99021 (1 μM). The relative gene expression levels showed that most cyclin genes were significantly upregulated in cardiomyocytes treated with either N-cadherin antibody or CHIR99021. Error bars indicate s.d., *n* = 3 experiments. *P < 0.05 and **P < 0.01 for Kruskal-Wallis one-way analysis of variance compared to control

Earlier data demonstrated that the loss of N-cadherin seen in *AHF-Cre*; *Cdh2*^*fl/fl*^ mutant mice embryo causes lower levels of both membrane and nuclear β-catenin due to cytoplasmic degradation of β-catenin. Resultant downregulation of Wnt signaling led to the decrease in proliferation of CPCs, premature differentiation of AHF CPC to cardiomyocytes in the pharyngeal mesoderm, and disrupted growth of the OFT and RV [5]. As N-cadherin was found to be weakly expressed in proliferative CPCs and highly expressed in less proliferative cardiomyocytes, we therefore hypothesized that N-cadherin expression plays an important role in regulating the Wnt/β-catenin signaling pathway for proliferation and regeneration in mature cardiomyocytes. To test this hypothesis, we sought to re-activate Wnt signaling in matured mouse cardiomyocytes and assess their proliferation capabilities. Immunostaining for BrdU, a proliferation marker, was performed to assess proliferation of matured mouse cTnT+ cardiomyocytes. To affect availability of cytoplasmic β-catenin for nuclear translocation, we treated mouse cardiomyocytes with either N-cadherin antibody, which could potentially mimic Wnt signaling ligand binding or destabilize the adherens junction for the release of β-catenin, or CHIR99021, a GSK inhibitor. The results showed that more BrdU and cTnT double-positive cells were found in cardiomyocytes of either treatment, suggesting that upregulation of Wnt signaling via cytoplasmic β-catenin availability is able to enhance proliferation in matured mouse cardiomyocytes (Fig. 1a, b). These results were validated by quantitative PCR, whereby the cyclin genes known for their roles in cell cycle such as *CDK1*, *CCNA2*, *CCNB1*, and *CCNB2* were observed to be significantly upregulated in both N-cadherin antibody- and CHIR99021-treated cardiomyocytes, suggesting that both treatments successfully increased cytoplasmic β-catenin levels, reactivate Wnt signaling, and result in the matured mouse cardiomyocytes entering a more proliferative state (Fig. 1c).

### Neutralizing N-cadherin signaling leads to release and nuclear translocation of β-catenin and activation of Wnt signaling

**Fig. 2:**
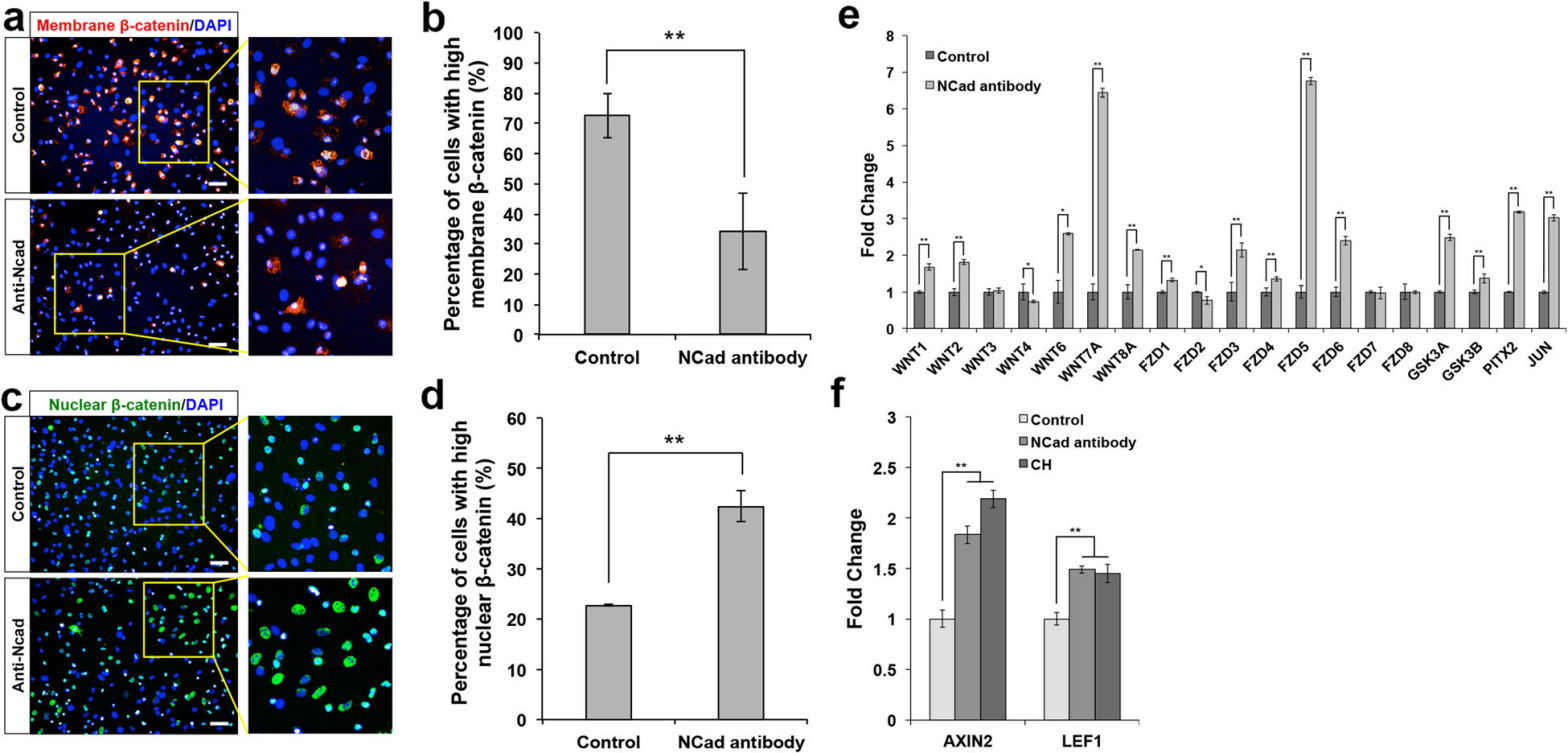
Neutralizing N-cadherin signaling results in translocation of β-catenin to the cell nuclei. Matured mouse cardiomyocytes were isolated via FACS using VCAM-1 antibody. **a** Representative images of untreated control and N-cadherin antibody (1:1000)-treated matured mouse cardiomyocytes immunostained for membrane-bound β-catenin (red). Cell nuclei (blue) were stained with DAPI. Scale bar, 50 μm. **b** Graphical representation of the percentage of cells with high membrane-bound β-catenin. A cut-off value > 150 was imposed to remove background. N-cadherin antibody-treated cardiomyocytes showed reduced amount of membrane-bound β-catenin (~ 50%) compared to the untreated control. Error bars indicate s.d., *n* = 3 experiments, **P < 0.01, evaluated by Student’s *t* test. **c** Representative images of untreated control and N-cadherin antibody (1:1000)-treated matured mouse cardiomyocytes immunostained for nuclear β-catenin (green). Cell nuclei (blue) were stained with DAPI. Scale bar, 50 μm. **d** Graphical representation of the percentage of cells with high nuclear β-catenin. A cut-off value > 300 was imposed to remove background. N-cadherin antibody-treated cardiomyocytes showed a 1-fold increase in the amount of nuclear β-catenin compared to the untreated control. Error bars indicate s.d., *n* = 3 experiments, **P < 0.01, evaluated by Student’s *t* test. **e** Quantitative PCR analysis of mRNA isolated from cardiomyocytes from untreated control and cardiomyocytes treated with N-cadherin antibody (1:1000). The relative gene expression levels showed that most genes in canonical Wnt signaling pathway were significantly upregulated in cardiomyocytes treated with N-cadherin antibody. Error bars indicate s.d., *n* = 3 experiments. *P < 0.05 and **P < 0.01, evaluated by Student’s *t* test. **f** Quantitative PCR analysis of mRNA isolated from cardiomyocytes in untreated control and cardiomyocytes treated with N-cadherin antibody (1:1000) or CHIR99021 (1 μM). The relative expression levels of Wnt signaling effector genes (*Axin2* and *Lef1*) were upregulated in cardiomyocytes treated with either N-cadherin antibody or CHIR99021. Error bars indicate s.d., *n* = 3 experiments. **P < 0.01 for Kruskal-Wallis one-way analysis of variance compared to control

While previous studies have reported that cadherins generally function as a negative regulator of Wnt/β-catenin signaling due to their role in sequestering cytoplasmic β-catenin at the cell membrane, they also play an important role in Wnt/β-catenin signaling during development [5, 20]. We investigated the changes of cytoplasmic and nuclear β-catenin levels after treatment with N-cadherin antibody to uncover the interaction between N-cadherin-based adherens junctions and the canonical Wnt signaling pathway. Immunostaining results and quantification of N-cadherin antibody-treated matured cardiomyocytes showed lower levels of membrane β-catenin but higher levels of nuclear β-catenin as compared to control (Fig. 2a–d). These results confirmed that there was translocation of β-catenin from the membrane to the nucleus upon treatment with N-cadherin antibody. We reasoned that N-cadherin antibody neutralizes N-cadherin signaling, preventing their ability to sequester β-catenin at the cell membrane which consequently leads to the release of membrane-bound β-catenin, resulting in larger reservoir of cytoplasmic β-catenin that undergoes nuclear translocation to activate Wnt signaling. Quantitative PCR results of these N-cadherin antibody-treated cardiomyocytes showed that many Wnt signaling-related genes were upregulated (Fig. 2e). We figured that treatment with GSK inhibitor, CHIR99021, should achieve Wnt activation through the same pathway due to the increased availability of cytoplasmic β-catenin levels. Therefore, we performed quantitative PCR on matured cardiomyocytes treated with N-cadherin antibody and the GSK inhibitor, CHIR99021, and observed upregulation of the main Wnt signaling effectors, *Axin2* and *Lef1* (Fig. 2f). Taken together, these results prove that neutralizing N-cadherin signaling results in high levels of cytoplasmic β-catenin for nuclear translocation to promote the re-activation of Wnt signaling in matured cardiomyocytes.

### Neutralizing N-cadherin signaling in *Cdh2* knockdown cells does not affect the Wnt signaling pathway

**Fig. 3:**
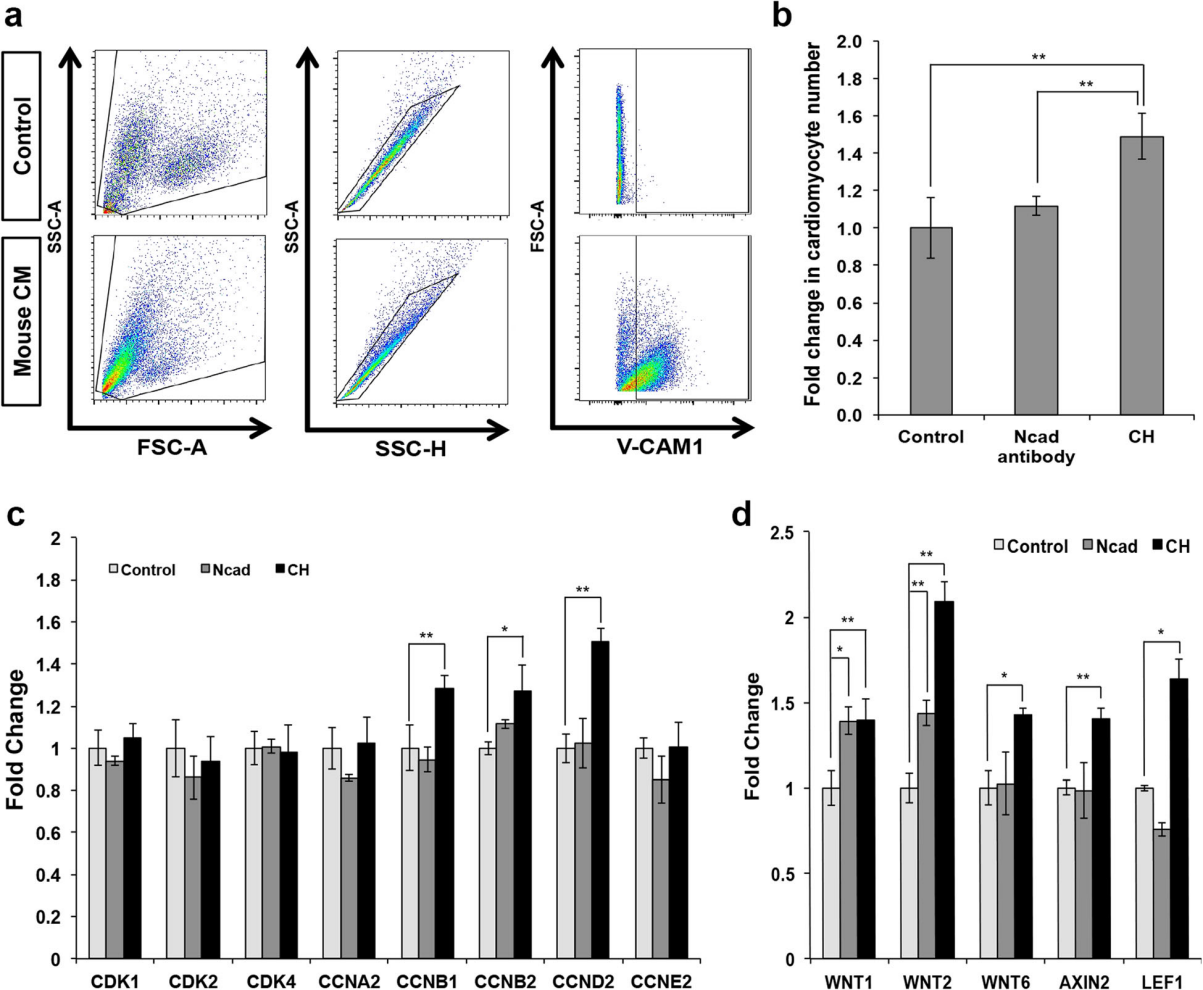
Cell proliferation only observed in CHIR99021-treated cardiomyocytes derived from *cdh2*^*−/−*^ mouse ES cells. **a** Representative FACS plots illustrating purification of VCAM-1-positive mouse cardiomyocytes. **b** Graphical representation showing fold change in cardiomyocytes proliferation in untreated control and cardiomyocytes treated with N-cadherin antibody (1:1000) or CHIR99021 (1 μM) normalized to control. Error bars indicate s.d., *n* = 3 replicates. **P < 0.01 for Kruskal-Wallis one-way analysis of variance compared to control. **c**, **d** Quantitative PCR analysis of mRNA isolated from cardiomyocytes in untreated control and cardiomyocytes treated with either N-cadherin antibody (1:1000) or CHIR99021 (1 μM). The relative gene expression levels showed that cyclin genes such as *Ccnb1*, *Ccnb2*, and *Ccnd2* were significantly upregulated only in cardiomyocytes treated with CHIR99021. Correspondingly, genes in canonical Wnt signaling pathway (*Wnt1*, *Wnt2*, and *Wnt6*) and effector genes (*Axin2* and *Lef1*) were shown to be significantly upregulated in cardiomyocytes treated with CHIR99021. Error bars indicate s.d., *n* = 3 replicates. *P < 0.05 and **P < 0.01 for Kruskal-Wallis one-way analysis of variance compared to control

To further support our hypothesis that neutralizing N-cadherin signaling results in increased proliferative capacity of matured mouse cardiomyocyte through the re-activation of Wnt signaling, we treated N-cadherin knockout mouse ES cell-derived cardiomyocytes with N-cadherin antibody or CHIR99021. The mouse ES cell-derived cardiomyocytes were first purified using fluorescence-activated cell sorting (FACS) based on their expression of vascular cell adhesion molecule-1 (V-CAM1) [21] (Fig. 3a); thereafter, the cardiomyocyte numbers between treatments and the control were compared (Fig. 3b). Significant increase in numbers of cardiomyocytes was observed only for CHIR99021 treatment, compared to the control, and as expected, N-cadherin antibody failed to have significant effects on cardiomyocytes derived from *Cdh2* knockout mouse ES cells. This result supports our earlier hypothesis that cardiomyocyte proliferation through N-cadherin antibody treatment was due to the release of sequestered β-catenin at the cell membrane, which undergoes nuclear translocation to activate Wnt signaling. Consistent with the above data regarding increased cell numbers and proliferation, we observed upregulation of genes involved in cell cycling (*Ccnb1*, *Ccnb2*, and *Ccnd2*) and Wnt signaling (*Wnt1*, *Wnt2*, *Wnt6*, *Axin2*, and *Lef1*) in CHIR99021-treated cardiomyocytes (Fig. 3c, d). While Ccnd2 activity is required for cell cycle G1/S transition, the family of cyclin B genes are essential mediators of cell cycle progression in the G2-M phase transition and promotes cell proliferation [22, 23]. In contrast, cell cycling genes were not significantly upregulated in N-cadherin antibody-treated cardiomyocytes. As cardiomyocytes derived from *Cdh2* knockout mouse ES cells do not form N-cadherin-based adherens junctions, our results confirm that N-cadherin antibody is only able to activate Wnt signaling and proliferation through interactions with N-cadherin.

### Directed differentiation and maturation of human ES cell-derived cardiomyocytes express a matured phenotype for modeling adult cardiomyocytes

**Fig. 4:**
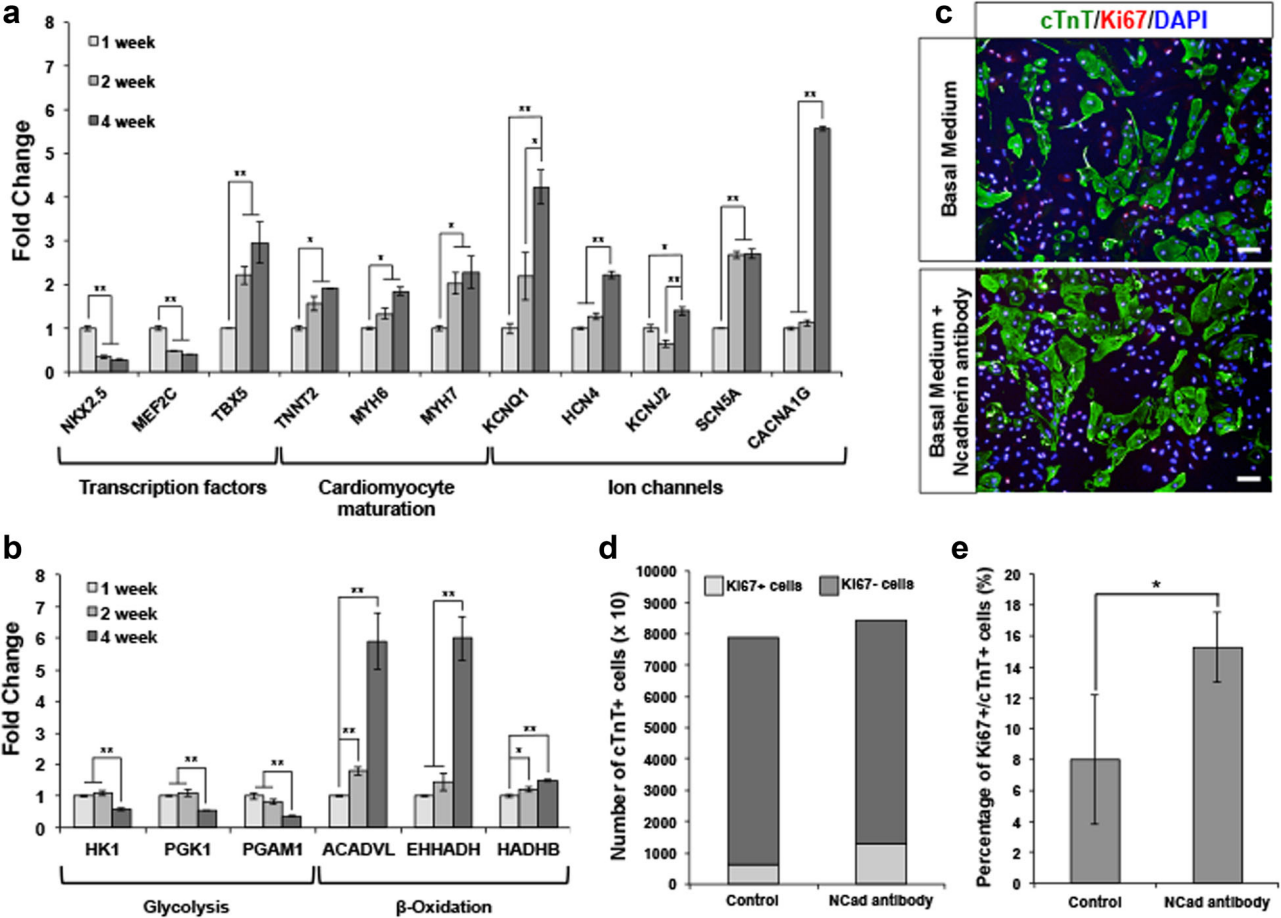
Quantitative PCR analysis of mRNA isolated from cardiomyocytes at various time points post-initial contraction (1, 2, and 4 weeks). Upregulation of Wnt signaling leads to proliferation of human ES cell-derived cardiomyocytes**. a** The relative gene expression levels showed downregulation of early key transcription factors governing cardiogenesis (*NKX2.5* and *MEF2C*) and upregulation of genes associated with cardiomyocyte maturation such as MYH7, *KCNQ1*, *SCN5A*, *HCN4*, and *CACNA1G*. **b** Concomitantly, metabolic shift from glycolysis to β-oxidation was observed. Error bars indicate s.d., *n* = 3 replicates. *P < 0.05 and **P < 0.01 for Kruskal-Wallis one-way analysis of variance compared to control. **c** Representative images of untreated control and N-cadherin antibody-treated human ES cell-derived cardiomyocytes immunostained for Ki67 (red) and cTnT (green). Cell nuclei (blue) were stained with DAPI. Scale bar, 50 μm. **d**, **e** Graphical quantification of the proportion of Ki67+ cells in N-cadherin antibody-treated human ES cell-derived cardiomyocytes compared to untreated cardiomyocytes (control). There is an increase of ~ 1.0-fold change of Ki67+/cTnT+ cells in N-cadherin antibody-treated cardiomyocytes compared to control. Error bars indicate s.d., *n* = 3 experiments, *P < 0.05, evaluated by Student’s *t* test

To assess possible applications for regenerative medicine in humans, we generated human ES cell-derived cardiomyocytes to investigate if induced proliferative effects observed in the mouse model could be replicated in human cell models. To ensure that the human ES cell-derived cardiomyocytes used expressed a matured phenotype sufficient to model adult cardiomyocytes, the cardiomyocytes used in this study were cultured for at least 2 months post-initial contraction [18]. We performed gene expression analysis on cardiomyocytes, which were cultured for up to 1 month post-initial contraction. The results revealed downregulation of early key transcription factors governing cardiogenesis such as *NKX2.5* and *MEF2C* and upregulation of cardiac maturation and ion channel markers such as *MYH7*, *KCNQ1*, *SCN5A*, *HCN4*, and *CACNA1G* at the end of the time-course experiment (Fig. 4a). Furthermore, consistent with other studies [18, 24], a concomitant shift in metabolism from glycolysis to β-oxidation was observed as the differentiated cardiomyocyte matures (Fig. 4b). Therefore, we concluded that the cultured cardiomyocytes used, 2 months post-initial contraction, are sufficiently mature to model adult cardiomyocytes.

### Upregulation of Wnt signaling leads to proliferation of human ES cell-derived cardiomyocytes

To investigate if the enhanced proliferative effect induced by upregulation of Wnt signaling could be replicated in adult human cardiomyocytes, we treated human ES cell-derived cardiomyocytes with N-cadherin antibody and CHIR99021 and immunostained for Ki67 as the marker for cellular proliferation. Initial testing with N-cadherin antibody (1:1000) showed promising results, with quantification of immunostaining results showing ~ 1.0-fold increase in Ki67+ population of cells in the cultured human ES cell-derived cardiomyocytes compared to a non-treated control (Fig. 4c–e). Thereafter, we varied concentrations of N-cadherin antibody and CHIR99021 to determine the optimal concentrations of both treatments that would result in the highest increase in Ki67+ population within the cardiomyocytes (Additional file 1: Figure S1A-B). The optimal concentrations were determined to be 1:1000 N-cadherin antibody and 1 μM CHIR99021, with further increased concentrations of either resulting in decreased cellular proliferation or increased cell death respectively (data not shown). Consistent with the effects of CHIR99021, quantitative PCR results showed that different concentrations of the GSK inhibitor do not significantly affect transcription of *CDH2* or *CTNNB1*, genes that are upstream of β-catenin signaling. On the other hand, dose-dependent upregulation of both downstream effectors *AXIN2* and *LEF1* was observed (Additional file 1: Figure S1C).

**Fig. 5:**
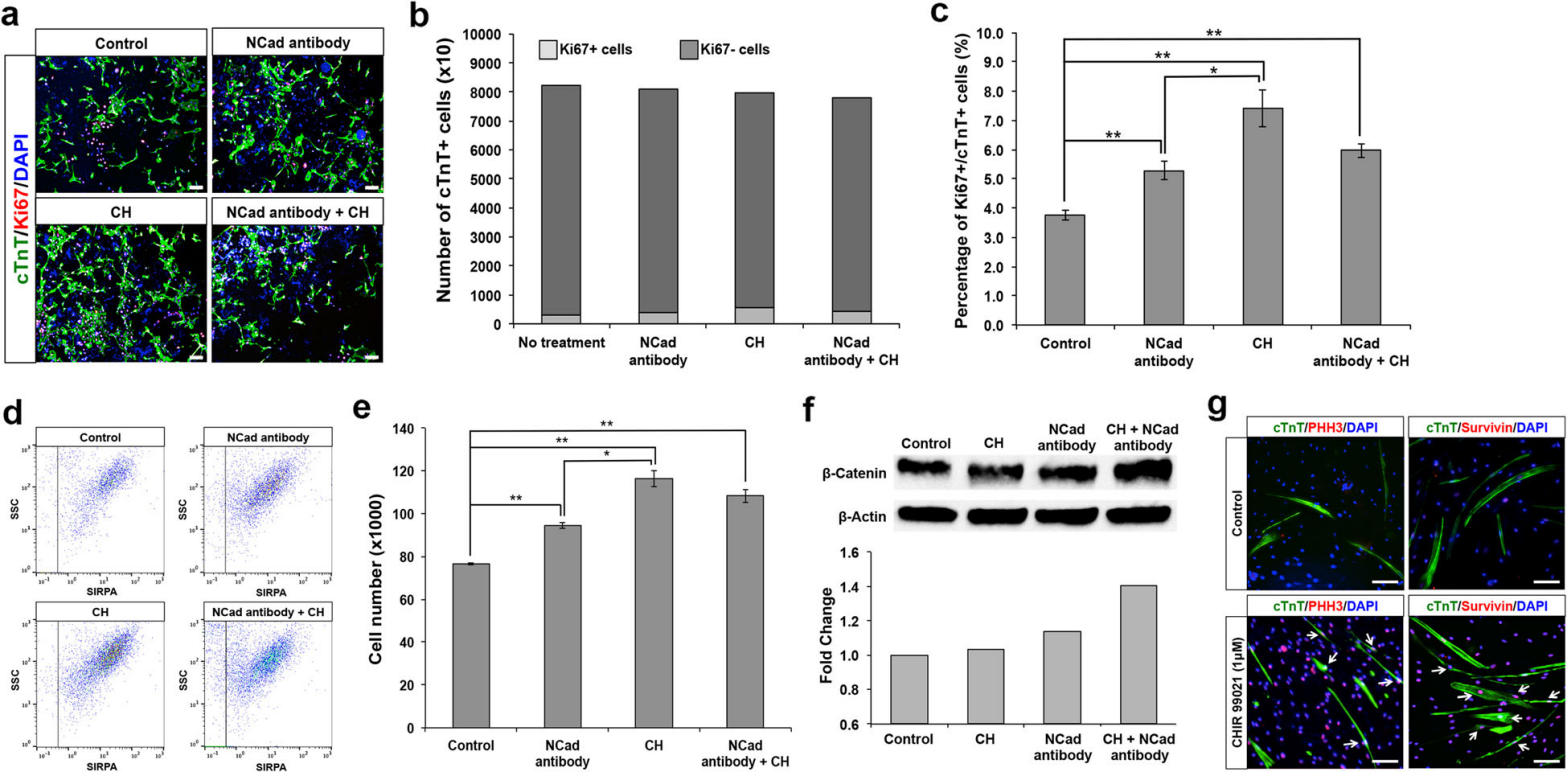
Wnt signaling activated proliferation variations between treatments of antibody, small molecule, or both. **a** Representative images of untreated human ES cell-derived cardiomyocytes (control) and treated human ES cell-derived cardiomyocytes with either N-cadherin antibody (1:1000), CHIR99021 (1 μM), or combinations of both N-cadherin antibody and CHIR99021 (1:1000; 1 μM) stained for cTnT (green) and Ki67 (red). Matured human ES cell-derived cardiomyocytes were enriched with SIRPA 2 months after initial contraction. Note the increased in number of Ki67+/cTnT+ cardiomyocytes treated with N-cadherin antibody, CHIR99021, or combinations of N-cadherin antibody and CHIR99021 as compared to control. Cell nuclei (blue) were stained with DAPI. Scale bar, 100 μm. **b**, **c** Graphical representation of the proportion of Ki67+ cardiomyocytes in respective culture conditions. All treated cardiomyocytes (cTnT+) resulted in an increase in proliferating (Ki67+) cells as compared to control. Error bars indicate s.d, *n* = 3 experiments. *P < 0.05 and **P < 0.01 for Kruskal-Wallis one-way analysis of variance compared to control. **d** Representative FACS plots illustrating SIRPA+ cardiomyocytes upon culture in various conditions (control, N-cadherin antibody (1:1000), CHIR99021 (1 μM), and N-cadherin antibody (1:1000) + CHIR99021 (1 μM)) for 7 days. **e** Graphical representation showing absolute number of cardiomyocytes determined via FACS in the various culture conditions. Error bars indicate s.d., *n* = 3 replicates. **P < 0.01 for Kruskal-Wallis one-way analysis of variance compared to control. **f** Immunoblot showing increased expression of cytoplasmic β-catenin when matured cardiomyocytes were cultured in the presence of CHIR99021 (1 μM), N-cadherin antibody (1:1000), or both. Cell lysates were probed with antibodies against cytoplasmic β-catenin and β-actin (loading control). Densitometry of band intensities was performed using ImageJ software. **g** Immunostaining of matured human ES cell-derived cardiomyocytes with either anti-PHH3 or anti-Survivin antibody after treatment with CHIR99021 (red). The results revealed that more cells undergo cytokinesis upon treatment with CHIR99021 (1 μM) as compared to the control. Cardiomyocytes were stained for cTnT (green). White arrow shows cardiomyocytes that were either cTnT+/PHH3+ or cTnT+/Survivin+. Cell nuclei (blue) were stained with DAPI. Scale bar, 100 μm

We then investigated if there are synergistic effects between N-cadherin antibody and CHIR99021 treatments. Immunostaining for Ki67+ and quantitative analysis on day 7 after treatment revealed that a combinatory treatment of N-cadherin antibody (1:1000) and CHIR99021 (1 μM) resulted in cardiomyocyte proliferation rates at an average between the more effective CHIR99021 treatment and the less effective N-cadherin antibody treatment (Fig. 5a–e). N-cadherin antibody treatment and CHIR99021 treatment resulted in a ~ 40% and ~ 100% increase in cardiomyocytes expressing the proliferation marker respectively, while the combinatory treatment resulted in a ~ 50% increase (Fig. 5b, c). Furthermore, flow cytometric analysis revealed that N-cadherin antibody and CHIR99021 both resulted in an increase in total cardiomyocyte number in culture compared to the control treatment, with the combinatory treatment also resulting in an average between the two treatments (Fig. 5d, e).

In order to demonstrate a direct correlation between unbound β-catenin in the cytosolic fraction with cardiomyocyte proliferation, we performed western blot on cytosolic fractions of control cardiomyocytes and cells treated with CHIR99021, N-cadherin antibody, or combinatory treatment for 1 day. Longer treatment duration was not preferred in this case as the release of membrane-bound β-catenin after treatment with N-cadherin antibody would not be observed if the lysates were collected at a later time point. Consistent with the results thus far, increased levels of unbound β-catenin compared to the control were observed after 1 day of treatment (Fig. 5f). As illustrated by the western blot results, stabilization of cytoplasmic β-catenin via CHIR99021 treatment results in its slow build-up for nuclear translocation, but the majority will still be sequestered at the plasma membrane due to high N-cadherin levels in matured cardiomyocytes. As expected, N-cadherin antibody treatment which acts as a ligand releases the larger pool of β-catenin observed (Fig. 5f), which is still susceptible to GSK-tagged degradation, possibly explaining the more significant upregulation of Wnt signaling effects from CHIR99021 treatment due to inactivated GSK. Finally, a dual treatment of CHIR99021 and N-cadherin antibody would therefore result in an even larger increase in cytoplasmic β-catenin, as the protein is both being released from the plasma membrane and being stabilized in the cytoplasm.

In addition to the upregulation of cytoplasmic β-catenin, more cardiomyocytes were stained positive for PHH3 and Survivin, a marker specific for G2-M phase that marks entry into the mitotic process and eventual division, when the cells were treated with CHIR99021 (Fig. 5g). All these results taken together suggest that both N-cadherin antibody and CHIR99021 induce dose-dependent cardiac proliferation through Wnt signaling and can possibly be used to revert mature human cardiomyocytes into a more proliferative state, up to a maximal dosage.

Despite that, we reasoned that excessive amounts of N-cadherin antibody hindered the formation of adherens junctions, inhibiting cell adhesion between cardiomyocytes leading to the loss of cardiomyocytes and inefficient proliferation. Sufficient amounts of free N-cadherin are required for proper cardiomyocyte proliferation, adhesion, and overall cardiac function. Similarly, we reasoned that high concentrations of CHIR99021 are cytotoxic due to the pleiotropic effects of GSK inhibitor which affects several other pathways. Overall, CHIR99021 at its optimal concentration exhibits the best potential as a therapeutic agent for cardiac regeneration as it promotes the maximal re-activation of Wnt/β-catenin signaling for cardiomyocyte proliferation without disrupting the necessary role of N-cadherin in cellular adhesion or inducing cytotoxicity.

### Wnt signaling upregulation without associated proliferation in cardiac injury models

Many known cardiopathies and injuries affect mainly the ventricles, causing inflammatory responses leading to scarring, fibrosis, and hypertrophy of the mammalian heart, referred to as ventricular remodeling. Wnt/β-catenin signaling has been implicated in cardiac remodeling regulation as an injury response. In the mouse model, numerous works have suggested Wnt/β-catenin signaling to play a part in regulating cardiac hypertrophy and preventing apoptosis. However, there has been little evidence uncovering the mechanisms and pathways involved. Wnt ligands have been shown to be upregulated alongside their feedback regulators Dkk1 and Dkk2 as a response in mouse models with induced cardiac injuries such as myocardial infarction [25–27].

In the current revision, we utilized human matured cardiomyocytes modeling cardiac injury to uncover how Wnt signaling is affected by cardiac injury. Cardiac hypertrophy has been induced as a form of heart injury in both animal and human models using oxidative stress treatments [28, 29]. Tert-butyl peroxide (TBP) is an oxidizing agent, commonly used as an alternative to hydrogen peroxide in stimulating oxidative stress in cells and tissues, while Endothelin-1 (EDN1) can result in cardiovascular fibrosis and hypertrophy due to increased collagen synthesis and reduced collagenase activity in cardiac fibroblasts [30, 31]. We treated human matured cardiomyocytes with either Endothelin-1 (EDN1) or Tert-butyl peroxide (TBP) to model cardiac injury and looked at changes in the transcript levels of *CDH2*, *CTNNB1*, *AXIN2*, and *LEF1*, effectors within the Wnt pathway (Additional file 1: Figure S2).

From the results, we observed that cardiomyocytes treated with EDN1 or TBP expressed reduced levels of *CDH2* transcription by ~ 0.5-fold while *CTNNB1* expression increased by ~ 2-fold. We hypothesize that diseased cells are undergoing a phenomena of dedifferentiation, thereby reducing surface N-cadherin and cell-cell contact and proliferation in an attempt to repopulate after cardiac injury. Dedifferentiation of cardiomyocytes for proliferation was first shown to occur in the naturally regenerating Zebrafish heart [32]. Several other researchers have also observed dedifferentiation when regeneration is induced in other models [33, 34]. We further investigated *AXIN2* and *LEF1* expression levels to understand this phenomena. *AXIN2*, being an immediate downstream target of β-catenin, shows a similar upregulation pattern to *CTNNB1* and serves as a negative feedback regulator of β-catenin. Interestingly, *LEF1*, the effector of proliferation signaling, is significantly downregulated upon TBP or EDN1 treatment. These results indicate that injured cardiomyocytes do not have a proliferative response despite the Wnt signaling received, suggesting that the upregulation of Wnt signaling via CHIR99021 treatment immediately after injury induced by ROS or EDN-1 may not result in cardiac regeneration in vivo. Nonetheless, CHIR99021 can be used in combination with simvastatin that has recently demonstrated to inhibit neural cell apoptosis and promote locomotor recovery via activation of Wnt signaling pathway by enhancing *Lef1* expression after spinal cord injury [35].

## Discussion

**Fig. 6:**
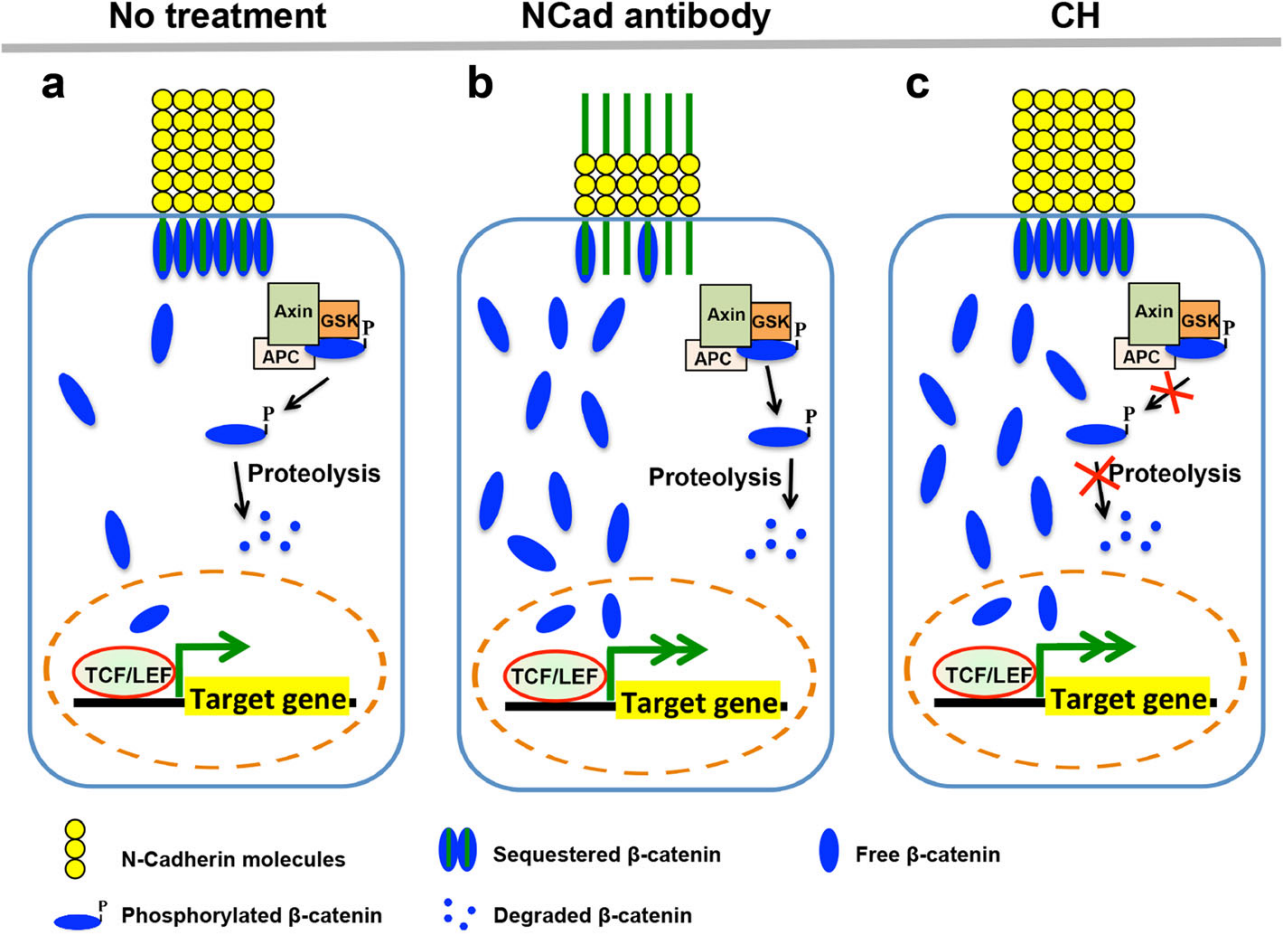
Schematic illustration of the mechanisms of Wnt/β-catenin signaling in untreated and treated (N-cadherin/CHIR99021) matured cardiomyocytes. **a** In untreated matured cardiomyocytes, the N-cadherin regulates the Wnt signaling by binding and sequestering cytoplasmic β-catenin at the cell membrane, hence reducing the amount of unbound cytoplasmic β-catenin that can be translocated into the nucleus. In addition, cytoplasmic β-catenin is also regulated by destruction complex, which will phosphorylate cytoplasmic β-catenin and targets it for ubiquitination and proteolysis. **b** Neutralizing N-cadherin signaling by N-cadherin antibody prevents the sequestration of β-catenin at the cell membrane which consequently leads to the release of membrane-bound β-catenin, resulting in a larger reservoir of cytoplasmic β-catenin that gets translocated into the nucleus to activate Wnt signaling for cardioproliferation. However, the function of N-cadherin in cell adherence is also disrupted by N-cadherin antibody treatment. **c** GSK inhibitor, CHIR99021, inhibits the function of β-catenin destruction complex and thus prevents the degradation of cytoplasmic β-catenin, resulting in a larger reservoir of cytoplasmic β-catenin that gets translocated into the nucleus to activate Wnt signaling for cardioproliferation. Note that CHIR99021 treatment does not affect cell adhesion, an important factor in cardioproliferation

Previous studies have shown that sustaining high Wnt signaling activity in AHF CPCs during early cardiogenesis promotes proliferation and prevents premature differentiation of CPC [5]. In this study, we further document the mechanism for the re-activation of quiescent Wnt signaling in matured mouse and human cardiomyocytes that leads to cardioproliferation by maintaining high levels of cytoplasmic β-catenin for nuclear translocation using N-cadherin antibody and GSK inhibitor, CHIR99021 (Fig. 6). Earlier studies have reported that N-cadherin sequesters cytoplasmic β-catenin at the cell membrane, preventing its proteolytic degradation by the proteasome complex while simultaneously preventing the β-catenin from exerting its Wnt signaling effect through nuclear translocation [5]. We demonstrated that neutralizing N-cadherin signaling results in an effect similar to Wnt ligand binding and release of membrane-bound β-catenin, causing an increase in Wnt signaling. Although cadherins generally function as a negative regulator of Wnt signaling, they play a crucial role in protecting a cytoplasmic store of β-catenin from proteolytic degradation which can be released upon Wnt ligand binding to activate the Wnt pathway. Confirmatory treatment of cardiomyocytes derived from *Cdh2* knockout mouse ES cells with N-cadherin antibody validated that the Wnt signaling upregulation in wild-type mouse treatments was solely due to the release of β-catenin sequestered and protected by proteolytic degradation by N-cadherin. However, it is important to note that N-cadherin also plays a role in cell adhesion which is essential in determining the cardioproliferation efficiency. We observed that concentrations of N-cadherin antibody beyond 1:1000 were less effective in inducing cardioproliferation as they cause cells to detach from each other leading to the loss of cardiomyocytes and inhibition of cell-cell adhesion necessary for proliferation.

While β-catenin/Wnt signaling is one such pathway that the cadherins regulate, there are several other proliferative and survival pathways that require N-cadherin/adherens junctions-mediated cell-cell adhesion. For instance, cadherin-mediated cell-cell contact is necessary for mechanical coupling between cells to activate mechanical signaling through both the actomyosin cytoskeleton and microtubule networks, shown to be crucial for cell division and transcriptional regulation [36, 37]. The closely related Notch and Hedgehog signaling pathways have also been shown to be regulated by N-cadherins in neural progenitor cells (NPCs). Notch1 and its ligand Dll1, distributed around adherens junctions, determine the apical polarity and guide asymmetric neurogenic division for NPCs and their differentiated progenies [38]. Alpha-catenin, which is also sequestered at the adherens junction, highly upregulates Hedgehog signaling leading to excessive NPC proliferation when deleted [39]. In skin tissue, α-catenin tightly regulates the activity of YAP1, the transcriptional effector of the Hippo pathway, which controls epidermal stem cell proliferation and tissue expansion [40]. Additionally, endothelial cells maintain cell-cell contact through molecular players such as the cadherins and gap junctions and have been shown to increase cell proliferation via PI3K-dependent signaling pathway [41]. In matured myocardium, continuous expression of N-cadherin is essential for normal cardiac electrophysiological activity due to their functions in gap junctions [42].

In our study, we demonstrated that CHIR99021 treatment exhibited the best potential for cardiac regeneration as it is not only more efficient in inducing cardioproliferation, it also does not interfere with the function of N-cadherin required for cell adhesion and cardiac electrophysiological activity [42]. Consistent with this, recent studies have demonstrated that CHIR99021 alone can promote cardiomyocyte proliferation in neonatal atrial human cardiomyocytes [43], emphasizing again the feasibility of using CHIR99021 as a potential drug that can endogenously repair injured cardiac tissue and promote cardiac regeneration. We found that CHIR99021 exhibits cytotoxic effects at a concentration of more than 1 μM, plausibly due to several reasons which were not uncovered in this study. GSK functions in several intracellular signaling pathways which result in CHIR99021, a GSK inhibitor to have several reported pleiotropic effects. One such pathway affected would be the nuclear translocation of transcription factor EB which leads to the activation of lysosomal/autophagy pathway, stimulating lysosomal biogenesis resulting in GSK inhibitor-induced apoptosis [44–46]. High concentrations of GSK inhibitor were also reported to cause centrosome aberrations, deformation in mitotic spindles, chromosome instability, and subsequently cell death from mitotic catastrophe [47].

While re-activation of Wnt signaling by CHIR99021 induces cardioproliferation required for cardiac regeneration, unregulated activation of Wnt signaling has been associated with many cardiac diseases such as cardiac hypertrophy and even cancer [48, 49]. While activated Wnt signaling can be useful in promoting cell proliferation, studies have also shown that constitutively activated β-catenin can result in the hyperactivation of Wnt signaling. Conditional inactivation of adenomatous polyposis coli (APC) which forms part of the β-catenin destruction complex and increased expression of β-catenin/Lef-1 fusion protein can lead to cell death by apoptosis in mammalian intestinal epithelia [50–52]. Collectively, the results from the usage of different concentrations of N-cadherin antibody and CHIR99021 observed in our experiment support the evidence that the cellular responses to Wnt/β-catenin signaling is dosage dependent [53, 54]. Apart from that, several other studies have also reported that the Wnt/β-catenin signaling has directional and graded activity across the ventricular wall to regulate cardiomyocyte proliferation, with decreasing gradient from the outer to the inner myocardium [9, 55–57]. Therefore, it is important to consider the spatiotemporal pattern of Wnt/β-catenin signaling when testing potential therapeutic agents such as CHIR99021 to improve cardiomyocyte proliferation in the future.

The current study focuses on laying the groundwork for the use of matured cardiomyocytes that were expanded in vitro for cell-based therapy. While the data showcases evidences that suggest plausible application for CHIR99021 to be utilized directly as an in vivo therapy for heart regeneration by stimulating endogenous cardiomyocytes to proliferate, we found it unwise due to reports of uncontrolled activation of the Wnt pathway causing potential deleterious and cancerous effects [48–51]. Until we can sustainably release CHIR99021 in a controlled and targeted manner in an injured heart, it would be challenging to demonstrate application of the findings in vivo.

## Conclusion

We have demonstrated in this study that activating Wnt signaling pathway by availing cytoplasmic β-catenin for nuclear translocation can enhance proliferation of matured cardiomyocytes. More importantly, we revealed that CHIR99021 is a promising candidate for the in vitro expansion of matured cardiomyocytes. The current study also provided evidence of dosage-dependent cellular responses to Wnt/β-catenin signaling using N-cadherin antibody and CHIR99021, providing a plausible avenue in the near future to activate Wnt signaling for endogenous cardiac regeneration using activators of canonical Wnt signaling pathway.

## Additional file

Additional file 1: **Figure S1.** Upregulation of Wnt signaling leads to proliferation of human ES cell-derived cardiomyocytes. **Figure S2.** Expression levels of Wnt signaling genes are affected in a cardiac hypertrophy model. **Table S1.** Lists of human and murine primer sequences used in this study. (DOCX 167 kb)

## Abbreviations

BSA: Bovine serum albumin
CH: CHIR99021
cTnT: Cardiac troponin T
EDN1: Endothelin-1
FACS: Fluorescence-activated cell sorting
hESC: Human embryonic stem cell
Ncad: N-Cadherin
PBS: Phosphate-buffered saline
TBP: Tert-butyl peroxide
