# Supplementary material for: Wnt/β-catenin-mediated signaling re-activates proliferation of matured cardiomyocytes
Source: Stem Cell Res Ther. 2018 Dec 7;9:338. doi: 10.1186/s13287-018-1086-8 (PMC6286613; doi:10.1186/s13287-018-1086-8)
Supplement: Supplementary file 1 — Figure S1. Upregulation of Wnt signaling leads to proliferation of human ES cell-derived cardiomyocytes. Figure S2. Expression levels of Wnt signaling genes are affected in a cardiac hypertrophy model. Table S1. Lists of human and murine primer sequences used in this study. (DOCX 167 kb) [file 13287_2018_1086_MOESM1_ESM.docx]

**Additional file 1**

**
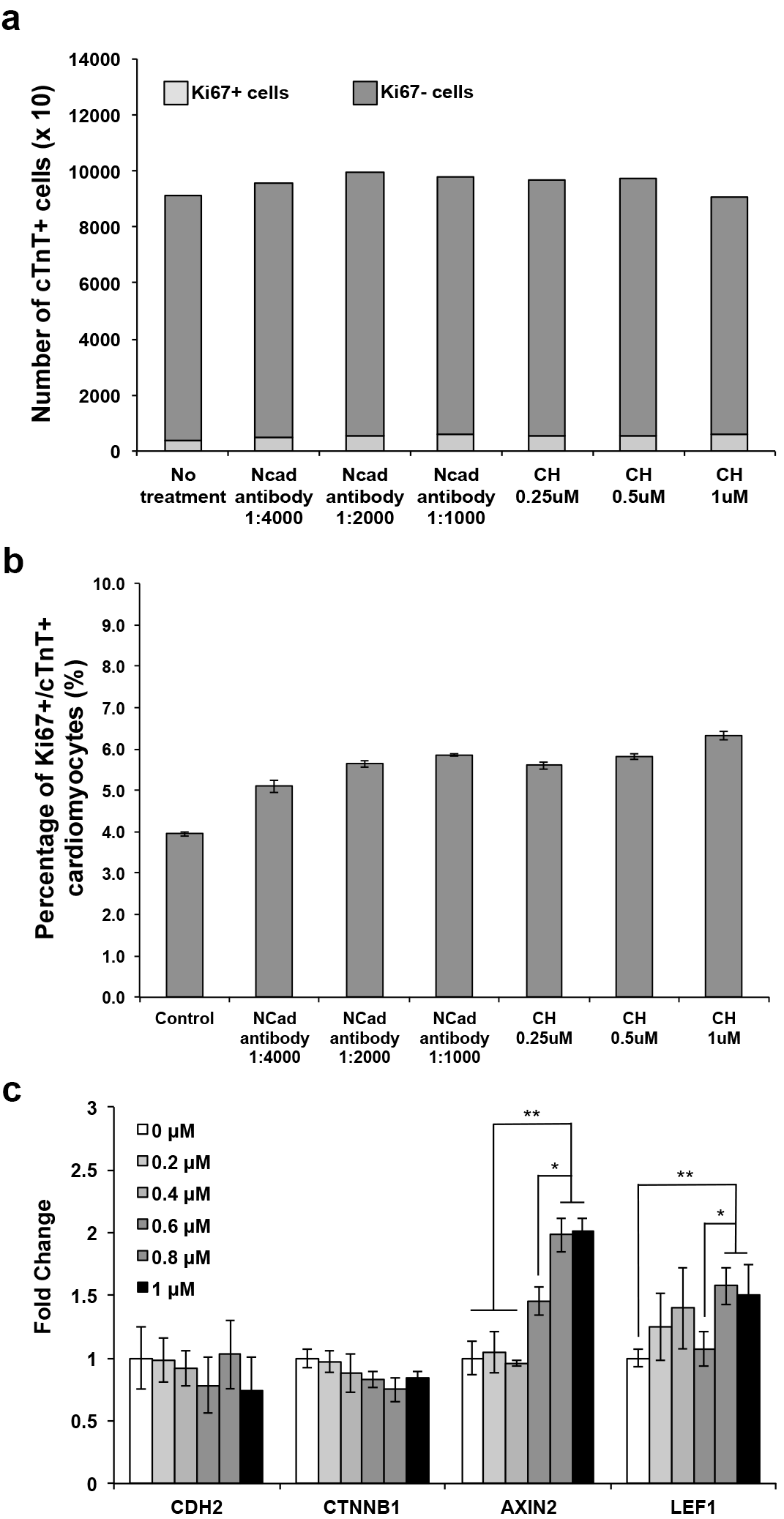
**

**Figure S1:** Up-regulation of Wnt signaling leads to proliferation of human ES derived cardiomyocytes. (A-B) Graphical quantification of the proportion of Ki67+ cardiomyocytes in different concentrations of N-cadherin antibody (1:4,000, 1:2,000 and 1:1,000) and CHIR99021 (0.25 µM, 0.5 µM and 1 µM) as compared to untreated cardiomyocytes (control). Note that the optimal concentrations of N-cadherin antibody (1:1,000) and CHIR99021 (1µM) treatments lead to ~50 % and ~60 % increase in Ki67 positive cardiomyocytes, respectively. (C) Expression levels of Wnt signaling genes of matured cardiomyocytes treated with different concentrations of CHIR99021. While *CDH2* and *CTNNB1* expression levels do not change significantly, *AXIN2* and *LEF1*, both downstream Wnt signaling effectors, were up-regulated with increasing concentrations of CHIR99021. Error bars indicated s.d, n = 3 replicates. * P < 0.05 and ** P < 0.01 for Kruskal-Wallis one-way analysis of variance compared to control.


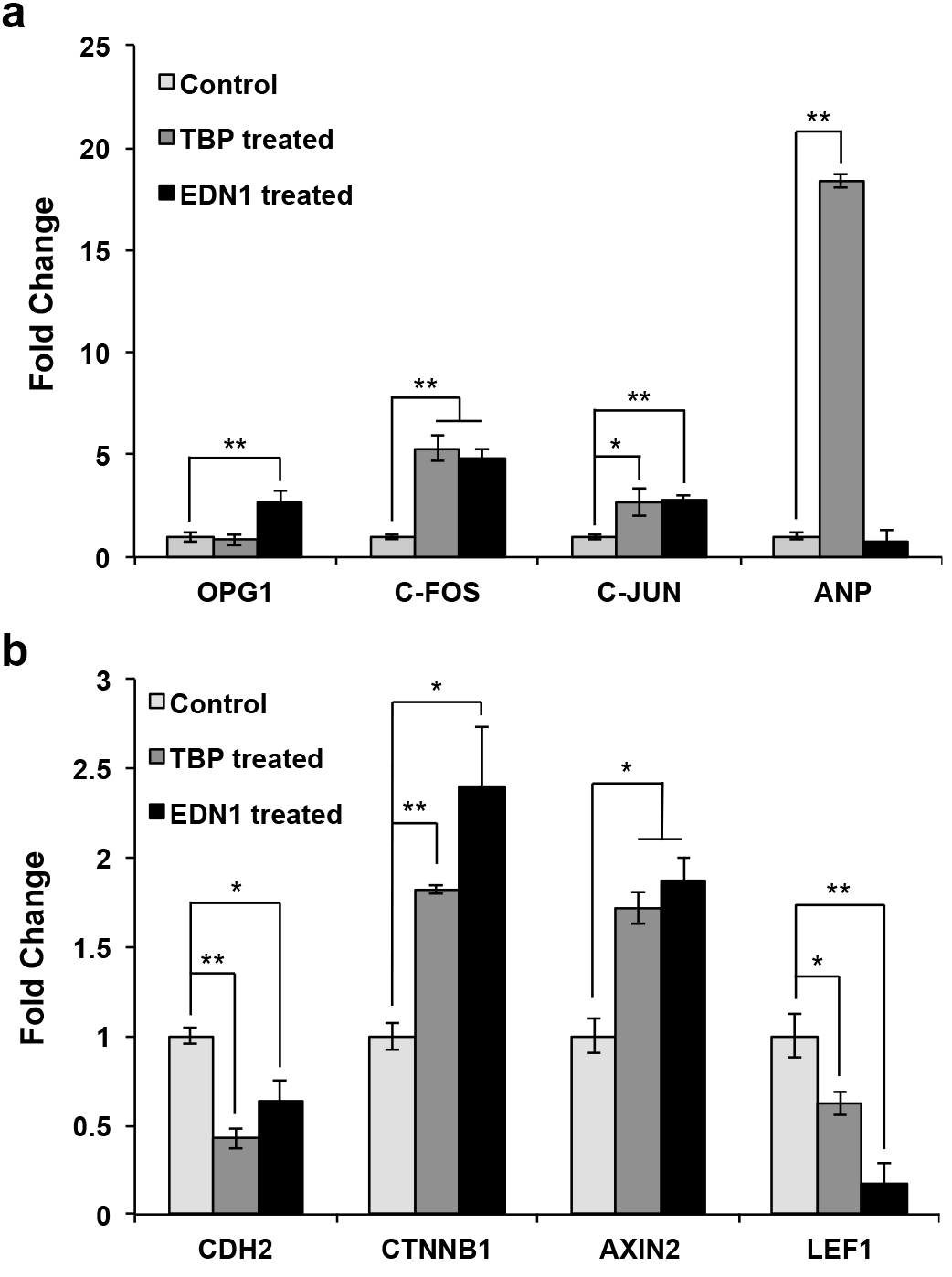


**Figure S2:** Expression levels of Wnt signaling genes are affected in a cardiac hypertrophy model. **(A)** Quantitative PCR analysis of mRNA isolated from cardiomyocytes of an injury model of cardiac hypertrophy. ES cell derived cardiomyocytes treated with Endothelin-1 (EDN1) and Tert-Butyl Peroxide (TBP) showed up-regulation of hypertrophy associated genes *OPG1*, *C-FOS*, *C-JUN* and *ANP*, relative to untreated control cardiomyocytes. Error bars indicated s.d, n = 3 replicates. * P < 0.05 and ** P < 0.01 for Kruskal-Wallis one-way analysis of variance compared to control. **(B)** Quantitative PCR analysis of *CDH2*, *CTNNB1*, *AXIN2* and *LEF1* transcript expression levels in cardiac hypertrophy model induced with either TBP or EDN1. Error bars indicated s.d, n = 3 replicates. * P < 0.05 and ** P < 0.01 for Kruskal-Wallis one-way analysis of variance compared to control.

**Table S1:** Lists of human and murine primer sequences used in this study.

| **Gene** | **Forward Primer** | **Reverse Primer** |
| --- | --- | --- |
| **CDK1** | TGGATCTGAAGAAATACTTGGATTCTA | CAATCCCCTGTAGGATTTGG |
| **CDK2** | AAAGCCAGAAACAAGTTGACG | GTACTGGGCACACCCTCAGT |
| **CDK4** | GTGCAGTCGGTGGTACCTG | TTCGCTTGTGTGGGTTAAAA |
| **CDK6** | TGATCAACTAGGAAAAATCTTGGA | GGCAACATCTCTAGGCCAGT |
| **CCNA2** | GGTACTGAAGTCCGGGAACC | GAAGATCCTTAAGGGGTGCAA |
| **CCNB1** | ACATGGTGCACTTTCCTCCT | AGGTAATGTTGTAGAGTTGGTGTCC |
| **CCNB2** | TGGAAAAGTTGGCTCCAAAG | TCAGAAAAAGCTTGGCAGAGA |
| **CCND1** | GCTGTGCATCTACACCGACA | TTGAGCTTGTTCACCAGGAG |
| **CCND2** | GGACATCCAACCCTACATGC | CGCACTTCTGTTCCTCACAG |
| **CCNE1** | GGCCAAAATCGACAGGAC | GGGTCTGCACAGACTGCAT |
| **CCNE2** | GCCATTGATTCATTAGAGTTCCA | CTGTCCCACTCCAAACCTG |
| **AXIN2** | GCTGACGGATGATTCCATGT | ACTGCCCACACGATAAGGAG |
| **LEF1** | CAGTCGACACTTCCATGTCC | GAGGGATGCCAGTTGTGTG |
| **ACTINB** | CCAACCGCGAGAAGATGA | CCAGAGGCGTACAGGGATAG |
| **FZD1** | cggcaagaccctcaactc | ccttgtttgctgttggtgag |
| **FZD2** | caccccaccaggagtactga | acaagtgctcaaaggcgaat |
| **FZD3** | acagcaaagtgagcagctacc | ctgtaactgcagggcgtgta |
| **FZD4** | ttcacaccgctcatccagta | tgcacattggcacataaaca |
| **FZD5** | accccaggggagagaaac | tgcaaattgggggaagtaag |
| **FZD6** | cgtctatgagcaagtgaacagg | aattctggtcgagcttttgc |
| **FZD7** | gccagcttgtgcctaatagaa | agccgggagaaactcacag |
| **FZD8** | tgctctgcttcgtgtcca | aagcgctccatgtcgataa |
| **GSK3A** | gtctcctacatctgttctcgctact | cagccagctgaccaaacat |
| **GSK3B** | cccagaaccacctcctttg | ttgctgccgtccttgtct |
| **WNT1** | cgctggaactgtcccact | aacgccgtttctcgacag |
| **WNT2** | tttggcagggtcctactcc | cctggtgatggcaaatacaa |
| **WNT3** | ctcgctggctacccaattt | gagcccagagatgtgtactgc |
| **WNT4** | gcagagccctcatgaacct | cacccgcatgtgtgtcag |
| **WNT6** | cagttccagttccgcttcc | gaacacgaaggccgtctc |
| **WNT7A** | cttcgggaaggagctcaaa | gcaatgatggcgtaggtga |
| **WNT8A** | ccacaacaggctgagaagtg | tgctacagttcttggtgatgatg |
| **CDH2** | CCCAAGACAAAGAGACCCAG | GCCACTGTGCTTACTGAATTG |
| **CTNNB1** | GTTCAGTTGCTTGTTCGTGC | GTTGTGAACATCCCGAGCTAG |
| **JUN** | ccaaaggatagtgcgatgttt | ctgtccctctccactgcaac |
| **PITX2** | ccttacggaagcccgagt | ccgaagccattcttgcata |
| **OPG1** | GCTAACCTCACCTTCGAG | TGATTGGACCTGGTTACC |
| **C-FOS** | CACTCCAAGCGGAGACAGAC | AGGTCATCAGGGATCTTGCAG |
| **C-JUN** | TCCAAGTGCCGAAAAAGGAAG | CGAGTTCTGAGCTTTCAAGGT |
| **ANP** | CAGGATGGACAGGATTGGA | TGTCCTCCCTGGCTGTTATC |
| **Cdk1** | gaacttcgacatccaaatatagtcag | ccatggacaggaactcaaaga |
| **Cdk2** | cacagccgtggatatctgg | catggtgctgggtacacact |
| **Cdk4** | agagctcttagccgagcgta | ttcagccacgggttcatatc |
| **Ccna2** | cttggctgcaccaacagtaa | caaactcagttctcccaaaaaca |
| **Ccnb1** | gcgctgaaaattcttgacaac | ttcttagccaggtgctgcat |
| **Ccnb2** | caaccgtaccaagttcatcg | gagggatcgtgctgatcttc |
| **Ccnd2** | gctgtgcatttacaccgaca | acactaccagttcccactcca |
| **Ccne2** | gccatcgactctttagaatttca | tgtcatcccattccaaacct |
| **Wnt1** | acagtagtggccgatggtg | cttggaatccgtcaacaggt |
| **Wnt2** | cagagatcacagcctctttgg | gcgtaaacaaaggccgatt |
| **Wnt6** | gtgcaactgcacaacaacg | ggaacggaggcagcttct |
| **Axin2** | gagagtgagcggcagagc | cggctgactcgttctcct |
| **Lef1** | tcctgaaatccccaccttct | tgggataaacaggctgacct |
| **Gapdh** | cctgcttcaccaccttcttg | tgtccgtcgtggatctgac |
